# The young ones and the old: past & future of science

**DOI:** 10.1007/s12471-014-0578-0

**Published:** 2014-07-30

**Authors:** J. van der Velden, E. E. van der Wall

**Affiliations:** 1 Department of Physiology, Institute for Cardiovascular Research, VU University Medical Center, De Boelelaan 1117, 1081 HZ Amsterdam, the Netherlands; 2 Interuniversity Cardiology Institute of the Netherlands (ICIN) - Netherlands Heart Institute (NHI), PO Box 19258, 3501 DG Utrecht, the Netherlands

During recent years the scientific landscape has quickly changed due to technological advances and an explosion of ways to communicate. This new scientific landscape requires scientists to change their attitude from a mostly individualistic approach to collaboration within a group. The pace at which scientific research is performed has increased leading to a highly competitive environment characterised by the words innovative and excellence. To survive in this competitive world one needs to be multitalented and willing to invest time and energy, in particular at the start of one’s career. The optimal research career in the Netherlands requires publications in high-ranked journals and international training experience. However, one cannot stay abroad for too long, since the deadlines to apply for excellence grants (Veni innovation grant and Dekker programme of the Netherlands Heart Foundation) are strict and the young ones need to return to the Netherlands within a relatively brief period (2 to 3 years). The rather strict system was highlighted in the article ‘Science on the move’ [1], which illustrates that many Dutch people work abroad and may never come back to the Netherlands, as after several years they no longer fit into the career structure of the Netherlands. We should realise that this rather rigid system may discourage excellent young researchers from pursuing their career in the Netherlands. Cardiovascular training systems in the Netherlands and USA are not complementary as fellowships of the American Heart Association provide funding for a 5-year research project; a person who stays in the USA for this period would not be able to apply for the initial excellence grants in the Netherlands as they could not comply with the deadlines. Surviving in research is even more complex for the young ones in the clinic as they are supposed to keep their scientific output at an excellent level while doing their clinical training.

Although it appears that one has to be a super (wo) man, there are not only negative sides to this changing world. Nowadays, research can be done in a stimulating environment within national and international consortia, in which young people have the opportunity to discuss their science with multiple researchers who all have their own unique way of thinking. Due to collaborative research efforts, pathological mechanisms of disease can be studied from the molecular level to the patient. This not only requires a different kind of education, but also other social and communicative skills from young individuals who favour a career in science. Thus, the training of young scientists should be reorganised [2, 3]. Building your career track differs from the past and requires a slightly different tool kit.

Young ICIN is a group of talented young scientists from the clinical and preclinical arena. A year ago, they joined forces in order to think of strategies to train and nurture the next generation of cardiovascular researchers. In coming years, Young ICIN will set up a scientific network of young researchers with clear national and international visibility. Our overall mission is to secure the future of excellent cardiovascular research in the Netherlands.

What does this mean for the Netherlands Heart Journal? It almost naturally follows that a substantial number of Young ICIN members will be directly connected to NHJ. It was thought appropriate to involve them as associate editors, as it has been shown that young scientists are usually more ambitious about writing scientific papers and that they are sometimes even better reviewers than their seniors [4, 5]. We expect a lot from them. This also means that we have to say farewell to many members of the Editorial Board, not just because they are ‘too old’ but they have largely served their term; everyone has an initial term of two years to be renewed once. All the members we have to say goodbye to have crossed the four-year line. We thank them wholeheartedly for their contributions to our journal over the years.

As a result, the subdivision Associate Editors has been added to the Editorial Board structure, in which we have followed the policy of the European Heart Journal. As a next step, we have simplified the article categories to six main types of manuscripts: original articles, review articles, heart beats, editorial comments, rhythm puzzles, and letters to the editor. Heart Beat is a new category, replacing case reports and images. The freshly adapted Instructions for Authors will make this clear. Lastly, NHJ will engage in composing webcasts in conjunction with our educational institute, the CVOI, and our national society, the NVVC. All these challenges will broaden the scope of NHJ. To bring these challenges into reality, we have to rely on both the experience of the old generation and the eagerness of the young generation.
